# Role of Exosomes in Cancer and Aptamer-Modified Exosomes as a Promising Platform for Cancer Targeted Therapy

**DOI:** 10.1186/s12575-024-00245-2

**Published:** 2024-05-27

**Authors:** Yating Wu, Yue Cao, Li Chen, Xiaofeng Lai, Shenghang Zhang, Shuiliang Wang

**Affiliations:** 1 Fujian Key Laboratory of Aptamers Technology, Affiliated Dongfang Hospital of School of Medicine, Xiamen University, Fuzhou, Fujian Province P. R. China; 2 Department of Medical Oncology, Fuzhou General Clinical Medical School (the 900th Hospital), Fujian Medical University, Fujian Province, Fuzhou, P. R. China; 3 Department of Clinical Laboratory Medicine, Fuzhou General Clinical Medical School (the 900 th Hospital), Fujian Medical University, Fujian Province, Fuzhou, P. R. China

**Keywords:** Extracellular vesicles (EVs), Exosome, Cancer, Aptamer, Target therapy

## Abstract

Exosomes are increasingly recognized as important mediators of intercellular communication in cancer biology. Exosomes can be derived from cancer cells as well as cellular components in tumor microenvironment. After secretion, the exosomes carrying a wide range of bioactive cargos can be ingested by local or distant recipient cells. The released cargos act through a variety of mechanisms to elicit multiple biological effects and impact most if not all hallmarks of cancer. Moreover, owing to their excellent biocompatibility and capability of being easily engineered or modified, exosomes are currently exploited as a promising platform for cancer targeted therapy. In this review, we first summarize the current knowledge of roles of exosomes in risk and etiology, initiation and progression of cancer, as well as their underlying molecular mechanisms. The aptamer-modified exosome as a promising platform for cancer targeted therapy is then briefly introduced. We also discuss the future directions for emerging roles of exosome in tumor biology and perspective of aptamer-modified exosomes in cancer therapy.

## Introduction

Living cell exports diverse components via secretion of extracellular vesicles (EVs) under physiological as well as pathophysiological conditions [1]. EVs are lipid-bilayer membrane nanoparticles enclosed with bioactive cargo including proteins, lipids, metabolites and nucleic acids derived from donor cells [2]. Based on their different ways of generation, EVs are broadly classified into two categories, exosomes and ectosomes [3]. Exosomes are EVs of endosomal origin with a size range of ~ 40 to 160 nm in diameter, while ectosomes are generated by the direct outward budding of the plasma membrane which vary in size from ~ 50 to 1000 nm [4]. During the past decades, EVs have been increasingly recognized as important mediators of intercellular communication in cancer biology [5]. Through direct binding or uptake by cancer cells and cells in the tumor microenvironment (TME), EVs can elicit multiple biological effects to impact hallmarks of cancer such as metastasis and resistance to therapy [6, 7]. In addition to their role in cancer initiation and progression, EVs have also been attractively exploited to develop novel EV-based treatment of cancer owing to their excellent biocompatibility and tropism of the tumor microenvironment [5, 8].

Aptamers are single-stranded DNAs or RNAs that can be obtained from a PCR-based in vitro selection strategy known as systematic evolution of ligands by exponential enrichment (SELEX) [9, 10]. Through folding into unique 3D structures, aptamers can specifically bind to a variety of targets with high affinity [11]. When exosome meets aptamer, an aptamer-guided exosome has been extensively explored as a promising platform for cancer targeted therapy in the past decades [12, 13]. In this review, we summarize the current knowledge of roles of exosomes in risk and etiology, initiation and progression of cancer, as well as their underlying molecular mechanisms. The aptamer-modified exosome as a promising platform for cancer targeted therapy is then briefly introduced. We also discuss the future directions for emerging roles of exosome in tumor biology and perspective of aptamer-modified exosomes in cancer therapy.

## Biogenesis and Biological Properties of Exosomes

Our recognition of membrane-enclosed vesicles can be dated back to the late of 1960s [14]. In 1987, the term exosome was firstly proposed by Johnstone et al. to designate EVs of endosomal origin derived from in vitro cultured sheep reticulocytes [15]. Thereafter, tremendous efforts have focused on unravelling the mechanisms underlying biogenesis of exosome which have been well reviewed [1, 16].

To our current knowledge, the biogenesis of exosomes is initiated by endocytosis of molecular cargo into the cell to generate intraluminal vesicles (ILVs) [17]. The sorting of cargo is regulated by either an ESCRT (endosomal sorting complex required for transport)-dependent or ESCRT-independent manner [18–21]. The ESCRT is a multiprotein machinery consisting of four distinct assembled complexes: ESCRT-0, ESCRT-I, ESCRT-II, and ESCRT-III [22]. At the beginning of ESCRT-dependent biogenesis of ILVs, Vps27/Hrs, component of ESCRT-0, is localized to the early endosome and recruits ESCRT-I via binding of Vps 23/TSG101 [23–25]. Then, ESCRT-II is recruited to ESCRT-I and triggers the assembly of ESCRT-III [26]. ESCRT-III functions as key regulator of inward budding of membrane, which results in ILVs generation [27, 28]. In addition to ESCRTs, several other proteins such as ALG-2 interacting protein X (ALIX) and G protein-coupled receptor 143 (GPR143) were also reported to be involved in this process [29, 30]. In 2008, a sphingolipid ceramide-dependent mechanism accounting for the generation of ILVs in a mouse oligodendroglial cell line was initially reported by Trajkovic et al. [20]. This study actually opened a new research field of ESCRT-independent biogenesis of ILVs, the mechanism of which has been continually unravelled [21, 31–33]. Some cargoes in early endosomes will be sorted out for rapid recycling [34]. ILVs with cargo not destined for recycling will commit to the endosomal maturation pathway by giving rise to multivesicular bodies (MVBs) [35]. Fusion of MVBs with autophagosomes or lysosomes will lead to degradation of cargoes within them; in other situations, those MVBs with matured exosomes will be docked to and fuse with plasma membrane, which result in releasing of exosomes via exocytosis [36, 37].

There is now evidence showing that exosomes originated from different cells share many common biological properties including similar structure and size, holding the potential to transport bioactive components between cells, and so on [2]. Although the cargoes of exosomes vary depending on types of donor cells and their physiological states, some proteins such as CD9, CD81, CD63, flotillin, TSG101, ceramide, and ALIX generally present in almost all exosomes of different origins [4]. These proteins have been recognized as biomarkers of exosomes based on which specific tracing, processing, isolation and identification methods have been developed and broadly used [38–40]. Secreted exosomes can be taken up by adjacent or distant (for circulating exosomes) recipient cells via endocytosis followed by cargo release [41]. Released cargo can then function as exogeneous regulator of cell phenotype [42]. In addition, ligands presented on the surface of exosomes can bind directly with their corresponding surface receptors on the recipient cell to activate the downstream signaling cascade [43, 44].

## Tumor Biology of Exosomes

The initiation and progression of cancer is a complicate multi-step process involving ten widely accepted hallmarks that had been excellently proposed by Hanahan and Weinberg [7]. With the rapid progress in the analytical technique of exosome [45, 46], the dissection of the roles that exosomes play in the tumor biology has received much attention [5, 6]. Exosomes are now emerging as pivotal regulators of most if not all hallmarks of cancer [47].

### Role of Exosomes in Risk and Etiology of Cancer

It has long been known that in addition to the genetic and epigenetic compositions of individual, the contact of environmental etiology including chemical, physical as well as biological factors can also predispose victim to cancer [48]. Recently, cumulative evidence has demonstrated that exosome-mediated interactive communication between etiological factors and cells plays vital role in tumorigenesis [49–51]. A meta-analysis indicated that dairy product consumption may significantly increase the risk of diffuse large B-cell lymphoma (DLBCL), the most non-Hodgkin lymphoma (NHL) worldwide [52]. Mechanistic studies suggested that miR-148a, a miRNA enriched in milk-derived exosomes (MDEs) [53], may regulate the proliferation of B cell via simultaneously targeting DNA methyltransferase 1 (DNMT1) and TP53 [54, 55]. The cow’s milk consumption was also demonstrated to correlate with increased risk of estrogen receptor (ER)-positive/progesterone receptor (PR)-positive breast cancer by epidemiological evidence [56, 57]. Although the explanation to these findings is complicate, it is worth to note that the miRNAs in MDEs may play an important part via functioning as oncogenic factors in recipient cells in ER + breast cancer [50, 58].

The causative role of certain infectious pathogen in distinctive type of cancer is well established [59]. It was estimated that about 13% of new cancer cases could be attributable to infections in 2018 worldwide [60]. Among all carcinogenic infectious pathogens, *Helicobacter pylori (H. pylori*), high-risk human papillomavirus (HPV), hepatitis B virus (HBV), and hepatitis C virus (HCV) are the four most important pathogens, accounting for more than 90% of global infection-related cancers [61]. *H. pylori* is the most frequent cause of chronic gastritis [62], a pathology which leads to the development of around 90% of gastric cancer (GC) [63]. The mechanisms of infection of gastric epithelium by *H. pylori* predisposing patient to GC have been extensively explored [64]. CagA, a major virulence factor of *H. pylori*, influences many aspects of initiation of GC [65]. It has been reported that CagA is present in serum-derived exosomes in patient infected with *cagA*-positive *H. pylori*, suggesting that exosomes may act as novel mediators of carcinogenesis in GC [66–68]. Infection of Epstein–Barr virus (EBV) is related to a variety of human tumors of both lymphoid and epithelial origin, including Burkitt’s lymphoma, nasopharyngeal carcinoma (NPC) and GC [69]. Previous studies had confirmed that EBV-positive NPC cells could release exosomes containing latent membrane protein 1 (LMP1), the most important viral oncoprotein in NPC, and EBV-encoded microRNAs [70, 71]. The EBV product-containing exosomes were then shown to be transferred to uninfected recipient cells to activate oncogenic extracellular signal-regulated kinases (ERK) and v-Akt murine thymoma viral oncogene (AKT) signaling pathways [70]. More recently, a study by Lee et al. demonstrated that EBV could also remodel the microenvironment of NPC through activation of Yes-associated protein 1 (YAP1)/fibroblast activation protein-alpha (FAPα) signaling in fibroblasts mediated by exosome cargoes [72]. Chronic infection with HBV is one of the main risk factors for hepatocellular carcinoma (HCC) [73]. A variety of direct or indirect mechanisms underlying HBV-promoted HCC have been unravelled [74], among which exosomes are emerging as new important players in regulating hepatocellular carcinogenesis [51]. Cervical cancer, the fourth most frequently diagnosed cancer and the fourth leading cause of cancer death in women [75], is associated with high-risk human papillomavirus (HR-HPV) infection in 99.7% of cases [76]. There is increasing evidence indicating that exosomes derived from HPV-infected tumor cells carry genetic information of HPV and hold the capacity to reshape the immune landscape in cervical cancer [77–79]. Examples of exosomes involved in risk and etiology of cancer are provided in Table 1.

**Table 1:** Role of exosomes in risk and etiology of cancer

Exosomes source	Cargo	Key findings	Reference
Breast milk	miR-148a	Target DNMT1 to influence risk of cancer	[52–54]
Serum of patient	CagA	Establish a possible link between infection of H. pylori and development of GC	[66]
EBV-infected NPC cell	LMP1 and EBV-miRNAs	Activate oncogenic ERK and AKT signaling pathways in uninfected recipient cells	[70, 71]
HPV-positive cervical cancer cell	Viral DNA and transcripts	Provide evidence of exosome-mediated transfer of viral genetic information	[78, 79]

DNMT1: DNA methyltransferase 1; GC: gastric cancer; EBV: Epstein–Barr virus; LMP1: latent membrane protein 1; NPC: nasopharyngeal carcinoma; HPV: human papillomavirus

### Role of Exosomes in Initiation of Cancer

The malignant transformation of a normal cell is the first step of cancer initiation. In the experimental oncology, treatment with carcinogen results in random genetic changes in normal cells. Cells harbor oncogenic mutation(s) will be further transformed into tumor cells when receiving a second “hit”. It has been demonstrated that exosomes from human pancreatic cancer cells (PCs) but not normal cells could initiate transformation of NIH/3T3 cells by inducing mutations [80]. Exosomes derived from GC cells were also found to induce malignant transformation of normal gastric mucosa epithelium cells via transferring miR-15b-3p [81]. In an interesting study by Melo et al., pre-miRNAs and RISC-Loading Complex (RLC) including Dicer, AGO2, and TRBP were evidenced to be present in exosomes of breast cancer cells [82]. These exosomes exhibited cell-independent capacity to process precursor microRNAs (pre-miRNAs) into mature miRNAs and induce transcriptome alterations in recipient nontumorigenic epithelial cells and tumor formation in a Dicer-dependent manner [82].

The existing of cancer stem cells (CSCs) has been demonstrated by a series of landmark experiments in many common cancer types, including leukemia, breast cancer, colorectal cancer (CRC), and brain cancer [83, 84]. It has been widely accepted that the CSCs play essential role in initiation of tumor as well as recurrence, metastasis, and resistance to therapy [85]. The CSCs can be originated from the transformation of normal tissue stem cells [86]. In addition to the altered intrinsic signaling pathways, components of TME are increasingly recognized as indispensable regulators for the maintenance of CSCs [87]. There is mounting evidence indicating that TME-derived exosomes are involved in the regulation of CSCs in different types of cancers through delivering the long non-coding RNA (lncRNA) *H19* [88]. Recently, Zhuang et al. provided evidence demonstrating that cancer-associated fibroblasts (CAFs) could help to form a favorable niche to promote bladder cancer stemness via exosomes-mediated transfer of miR-146a-5p [89]. As key components of the TME in solid tumors, tumour-associated macrophages (TAMs) participate in regulating multiple aspects of tumorigenesis from genetic instability through to metastasis and tumour immunity [90]. It has been reported that TAMs can protect CSCs from cytotoxicity [91, 92]. While M1-like TAMs activated by exosome-transferred thrombospondin-1 (THBS1) have been demonstrated to promote malignant migration in oral squamous cell carcinoma (OSCC) by Xiao et al. [93], the same research group provided further evidence supporting that M1-like TAMs can cascade a mesenchymal/stem-like phenotype of OSCC via the IL6/Stat3/THBS1 feedback loop [94]. Several key findings of exosomes in initiation of cancer are summarized in Table 2.

**Table 2:** Role of exosomes in initiation of cancer

Exosomes source	Cargo	Key finding	Reference
PC cell	Undetermined	Initiate transformation of NIH/3T3 cells by inducing random mutations	[80]
GC cell	miR-15b-3p	Induce malignant transformation of normal gastric mucosa epithelium cell	[81]
BC cell	miRNAs and RLC	Induce transcriptome alterations in recipient nontumorigenic epithelial cell and transformation	[82]
Cancer cell	LncRNA *H19*	Regulate CSCs	*Reviewed in* [88]
CAFs	miR-146a-5p	Promote bladder cancer stemness by generating a favorable niche	[89]
OSCC cell	THBS1	Activate M1-like TAMs to promote malignant migration in OSCC	[93]
M1-like TAMs	IL-6	Cascade a mesenchymal/stem-like phenotype of OSCC	[94]

PC: pancreatic cancer; GC: gastric cancer; BC: breast cancer; RLC: RISC-loading complex; LncRNA: long non-coding RNA; CSCs: cancer stem cells; CAFs: cancer-associated fibroblasts; TAMs: tumour-associated macrophages; OSCC: oral squamous cell carcinoma; THBS1: thrombospondin-1; IL-6: interleukin-6

### Role of Exosomes in Progression of Cancer

After initiation, the mutated cell might remain indolent during the host’s lifespan [95]. Under certain condition, cancer cells can gain an outgrowth by acquiring the capabilities to sustain proliferative signaling, evade growth suppression, resist cell death, induce angiogenesis, as well as avoid immune destruction [7].

#### Exosomes-Mediated Regulation of Cell Proliferation

Dysregulated cell proliferation can be attributed to aberrant activation of mitogenic signaling achieved through a variety of alternative ways [96]. It has been comprehensively revealed that the receptor tyrosine kinases (RTKs)-regulated phosphoinositide 3-kinase (PI3K)/AKT signaling network controls most hallmarks of cancer including cell cycle [97, 98]. In glioma, a truncated and oncogenic form of the epidermal growth factor receptor (EGFR), known as EGFRvIII, was demonstrated to be present in microvesicles derived from tumour cells; these microvesicles can then transfer EGFRvIII to neighbour cells to activate downstream AKT signalling [99]. Similarly, exosomes derived from multiple cancer cell lines have been evidenced to induce mitogen-activated protein kinase (MAPK)-dependent monocyte survival through the transport of EGFR and human epidermal growth factor receptor-2 (HER2) [100]. Besides, studies also supported the notion that the autocrine and paracrine signals of cancer cells may achieved via an exosomes-mediated manner [101, 102]. Phosphatase and tensin homolog deleted on chromosome 10 (PTEN) plays a central role in counteracting PI3K/AKT signaling [103]. Cancer cells themselves have evolved many intrinsic mechanisms, including mutations, deletions, or transcriptional silencing, to gain loss of function of PTEN, thereby avoiding the negative feedback regulation of PI3K signaling by PTEN [104]. In addition, it has been demonstrated that CAFs-derived exosomes can promote the progression and chemoresistance of non-small cell lung cancer (NSCLC) by delivering miR-20 to target PTEN in recipient cells, suggesting that TME can also be harnessed by cancer cells to abrogate the inhibitory effect of PTEN on PI3K signaling [105]. Intriguingly, given the finding that PTEN itself can be exported in exosomes and exhibits phosphatase activity in recipient cells [106], it is rational that mutant PTEN may also be delivered in an exosomes-mediated manner.

#### Exosomes-Mediated Regulation of Cell Death

Regulated cell death (RCD) is central to maintain the homeostasis of multicellular organisms under physiological condition [107]. In contrast to accidental cell death (ACD), various modes of RCD including apoptosis, pyroptosis, ferroptosis, etc., have been characterized and each type can be triggered by distinct unrecoverable perturbations of the intracellular or extracellular microenvironment and manifest with an entire spectrum of morphological features [108]. Apoptosis is the firstly described type of RCD whose underlying mechanism has been fully elucidated. Cancer cells manage to avoid the destroy of intrinsic or extrinsic apoptosis through vary mechanisms [109]. The findings that exosomes derived from TAM hold the capacity to suppress cell apoptosis and enhance activation of PI3K/AKT signaling pathway by down-regulation of PTEN via transfer of miR-21 had shed new lights on role of exosome in resistance to apoptosis [110, 111]. The tRNA-derived fragments (tRFs) are a novel class of small non-coding ribonucleic acids (ncRNAs) derived from precursors or mature tRNAs, and their biological functions have received much attention in recent years [112]. In breast cancer, it has been demonstrated that exosome-transmitted tRF-16-K8J7K1B can reduce drug-induced cell apoptosis by targeting tumor necrosis factor-related apoptosis-inducing ligand (TRAIL) [113].

Pyroptosis is a type of RCD involving cellular defence mechanism against extracellular pathogen-associated molecular patterns (PAMPs) and damage-associated molecular patterns (DAMPs) in the canonical pathway or against intracellular LPS in the non-canonical pathway [114, 115]. While several studies indicated that the pyroptosis of non-cancer cells can be regulated by exosomes [116, 117], a recent investigation concluded that exosomes derived from highly metastatic melanoma tumor cells can transfer their metastatic competency to the low-metastatic melanoma tumor cells partially through exosomal miR-211-5p-regulated pyroptosis [118].

Ferroptosis was first described in 2012 involving the accumulation of lipid peroxides and oxidative stress leading to membrane damage [119]. As an iron-dependent form of RCD, the role of dysregulated ferroptosis in tumorigenesis has attracted much interest in recent years [120]. Through secreting exosomes, CAFs was demonstrated to inhibit ferroptosis in gastric cancer cells by miR-522-mediated targeting arachidonate lipoxygenase 15 (ALOX15) and blocking lipid reactive oxygen species (ROS) accumulation and induce chemoresistance [121]. Not coincidentally, adipocyte-derived exosomal microsomal triglyceride transfer protein (MTTP) was also evidenced to suppress ferroptosis and promote chemoresistance in CRC [122]. On the contrary, however, exosome‑mediated miR‑144‑3p has been verified to promote ferroptosis in osteosarcoma via regulating zinc-finger E-box-binding homeobox 1 (ZEB1) [123]. Thus, the precise role of ferroptosis in tumor biology depends on tumor type as well as the crosstalk between tumor and TME.

#### Exosomes-Mediated Regulation of Angiogenesis

Induced angiogenesis is essential for fueling the cancer with nutrients and oxygen as well as enabling it to evacuate metabolic wastes and carbon dioxide [7, 124]. The vascular endothelial cells (ECs) and smooth muscle cells (SMCs) are endowed with mechanisms to sense O2 supply in tissue [125]. In a solid cancer, O2 concentrations vary substantially owing the rapid cell proliferation. It has been well established that the hypoxia-induced angiogenesis is tightly regulated by the balance between proangiogenic and anti-angiogenic signals [126]. A substantial body of evidence indicates that hypoxia-inducible factors (HIFs) are broadly expressed in human cancer and play central role in switching angiogenesis [127]. HIF1, a heterodimer of HIF1α and HIF1β, is an important mediator of the hypoxic response of tumor cells and controls the up-regulation of a number of factors important for solid tumor expansion including the angiogenic factor vascular endothelial-derived growth factor (VEGF) [128]. The expression of HIF1α is subjected to both transcriptional and post-translational regulation [127]. Xia et al. reported that on the one hand, hypoxia could promote the expression of miR-301a-3p and release of miR-301a-3p-enriched exosomes by GC cells; on the other hand, exosomes can then transfer miR-301a-3p to recipient GC cells to inhibit HIF-1α degradation through targeting prolyl-hydroxylase 3 (PHD3) [129]. Hypoxia-induced release of exosomes was also evidenced in other cancer types including breast cancer [130].

The VEGF family includes VEGFA, VEGFB, VEGFC, VEGFD, VEGFE, and placental growth factor (PlGF), among which VEGFA plays a key role in regulating angiogenesis during homeostasis and disease [131]. Upon binding with its dominant signaling receptor VEGFR2 on endothelial cells, VEGFA triggers the canonical multiple downstream signaling cascades to regulate the proliferation, filopodial extension, chemotaxis and extracellular matrix (ECM) degradation [132]. Two separated studies demonstrated that exosomes derived from human umbilical vein endothelial cells (HUVECs) or CAFs could promote angiogenesis of HCC or CRC, respectively, through delivering VEGFA [133, 134]. Through RNA sequencing, the increased expression of a circular RNA, circSHKBP1, was found in tumor tissues and serum exosomes of patients with GC [135]. Mechanistically, circSHKBP1 was further revealed to promote angiogenesis by targeted regulating the miR-582-3p/HUR/VEGF axis [135]. In addition to circular RNA, exosomal miRNAs such as miR-30b-5p [136], miR-205 [137], miR-155-5p and miR-221-5p [138] were also demonstrated to promote angiogenesis of cancer through different mechanisms. Inconsistently however, there is study indicated that mesenchymal stem cell (MSC)-derived exosomes can suppress in vitro angiogenesis through modulating the mTOR/HIF-1α/VEGF signaling axis in breast cancer cells in an miR-100-dependent manner [139]. Collectively, it is worthy of mention that the role of exosomes in regulating angiogenesis is complex and highly context dependent, varying by their origin as well as the bioactive cargoes they carry [140].

#### Exosomes-Mediated Metabolic Reprogramming in Cancer

To fuel the cell growth and division, many aspects of cancer metabolism including uptake of glucose and amino acids are reprogrammed as well reviewed by Pavlova et al. [141]. Cancer cell tends to reprogram energy metabolism from oxidative phosphorylation (OXPHOS) to aerobic glycolysis even under sufficient oxygen conditions, a phenomenon termed Warburg effect which was first described by Otto Warburg nearly a century ago [142]. Although the Warburg effect has been extensively investigated, the detailed mechanisms triggering this effect during tumorigenesis is still being elucidated. As one of the most commonly activated pathways in human cancers, the aberrant PI3K/AKT signaling pathway resulted from diverse alterations such as Ras mutation has multiple downstream effects on cellular metabolism including glycolysis, through either direct regulation of glucose uptake or key steps in glycolysis via the phosphorylation and activation of specific glycolytic enzymes [143, 144]. There is a growing body of studies indicating that the crosstalk between cancer cells and TME mediated by exosomes can reprogram cancer metabolism via regulating PI3K/AKT signaling pathway [145, 146]. In a recent study, exosomes derived from bone marrow MSCs loading with pyrroline‑5‑carboxylate reductase 1 (PYCR1)-targeted siRNAs was showed to inhibit aerobic glycolysis of bladder cancer cells via regulation of the EGFR/PI3K/AKT pathway [147]. The lncRNA colon cancer-associated transcript-1 (CCAT1), firstly identified in CRC [148], is increased expressing in GC cells and participates in promoting the malignant progression of GC through enhancing autophagy [149]. Zhang et al. presented new evidence indicating that the expression of lncRNA CCAT1 was also significantly elevated in the tissues and plasma exosomes of patients with GC; moreover, lncRNA CCAT1 was confirmed to interact directly with polypyrimidine tract binding protein 1 (PTBP1) and effectively maintain its stability by inhibiting the ubiquitin-mediated degradation process, thus to facilitate the transition from PKM1 to PKM2 to augment glycolysis in GC cells [150]. It is worthy of note that the reprogrammed Warburg effect can in turn regulate many hallmarks of cancer through promoting the release of exosome by cancer cells [151, 152].

CAFs are the most common cell type in TME whose biological function in cancer metabolism has been extensively investigated [153]. First proposed by Pavlides et al. in 2009 [154], the “Reverse Warburg Effect” was recognized as epithelial cancer cells induced the aerobic glycolysis in neighboring stromal fibroblasts. In this condition, metabolic reprogrammed CAFs can then provide metabolites such as lactate for cancer cells and facilitate their proliferation through the tricarboxylic acid (TCA) cycle and OXPHOS [155, 156]. CAFs can originate from three major routes, among which the endothelial to mesenchymal transition (EndMT) has been demonstrated to be induced by cancer-derived exosomes [157]. Through secreting exosomal miR-105, breast cancer cells were demonstrated to enhance glycolysis in CAFs [158]. Breast cancer cells-derived exosomes were also evidenced to induce mitophagy and glycolysis in CAFs via delivering integrin beta 4 (ITGB4) [159]. Reciprocally, CAFs can also ingest cancer-derived lactate to maintain a fibrotic and immunosuppressive microenvironment in pancreatic ductal adenocarcinoma (PDAC) [160], suggesting an even more complicate model in term of metabolic reprogramming.

In addition to glucose, lipids are also important metabolites for the synthesis of biological membranes and signaling molecules required for fueling the rapid proliferation of cancer cell [161]. The lipid metabolism includes lipogenesis, lipid uptake, fatty acid oxidation, and lipolysis. Previously, much attention has been paid on the role of lipid metabolism in regulation of exosome biogenesis; however, exosomes-regulated lipid metabolism in tumorigenesis and cancer progression are emerging in recent years [162]. The expression of HBV pre-S2 trans-regulated protein 3 (HSPC111) was elevated in CRC cells with highly metastatic potential [163]. Through transmitted by exosomes, CRC cells-derived HSPC111 was demonstrated to alter lipid metabolism of CAFs by phosphorylating ATP-citrate lyase (ACLY), thus leading to the increased levels of acetyl-CoA. Accumulated acetyl-CoA functioned as an epigenetic regulator to promote the expression and secretion of CXCL5, which resulted in CRC cells colonized in liver via the CXCL5-CXCR2 axis [163]. Carnitine palmitoyltransferase 1 A (CPT1A) as a key enzyme in fatty acid oxidation (FAO) whose deregulation has been reported to be associated with grade, pathological stage, lymph node metastasis and poor prognosis in patients with GC [164]. A recent interesting study uncovered that a microprotein (pep-AKR1C2) encoded by exosomal lncAKR1C2 derived from GC cells can upregulate the expression of CPT1A in lymphatic endothelial cells via regulating YAP phosphorylation, thus promoting gastric cancer lymph node metastasis by enhancing FAO and ATP production [165]. Through a systematic analysis of gene expression profile in exosomes derived from PCs with different gemcitabine sensitivity by mass spectrometry, a total of 155 proteins were identified as differentially expressed between gemcitabine-resistant and -sensitive groups [166]. The KEGG pathway analysis revealed that the differentially expressed proteins were significantly enriched in metabolic pathways such as the TCA cycle, OXPHOS, and fatty acid metabolism. Furthermore, medium-chain acyl-CoA dehydrogenase (ACADM), an enzyme catalyzing the first step of FAO (β-oxidation) [167], was demonstrated to promote the gemcitabine-resistance in PCs via increasing hydrolysis of medium-and long-chain fatty acids [166]. Also of note, exosomal ACADM (Exo-ACADM) was strongly correlated with gemcitabine sensitivity in vivo, suggesting that it can be served as a predictor for postoperative gemcitabine chemosensitivity in patients with PC [166, 168].

Amino acids (AAs) act as not only the raw materials for cellular synthesis of biomolecules but also energy source. Glutamine (Gln), one of the most abundant nonessential AAs, participates in many fundamental biological processes such as energy formation, redox homeostasis, macromolecular synthesis, and signaling in cancer cells [169]. In cancerous tissue, cancer cells showed the highest uptake of glutamine as compared with other cell subsets in the TME, as evidenced by positron emission tomography (PET) tracers [170]. In the context of tumorigenesis, the uptake of glutamine by cancer cells can be promoted by exosomes derived from various types of cellular components in the TME through different mechanisms [171]. Zhang et al. discovered that M2 macrophage-derived exosomal miR-193b-3p enhanced the proliferation, migration, invasion, and glutamine uptake of PCs by targeting tripartite motif (TRIM)-containing protein TRIM62, resulting in the decrease of c-Myc ubiquitination [172]. CAFs were also demonstrated to regulate the glutamine metabolism in lung adenocarcinoma (LUAD) cells in an exosome-dependent manner [173]. In this study carried out by Liu et al., the lncRNA LINC01614 was showed to be upregulated in CAFs stimulated by tumor-derived proinflammatory cytokines and packaged into exosomes. Exosomal LINC01614 could directly interact with annexin A2 (ANXA2) and p65 to facilitate the activation of nuclear factor kappa-B (NF-κB), which led to the upregulation of the glutamine transporters SLC38A2 and SLC7A5 and eventually enhanced the glutamine uptake in LUAD cells [173]. Moreover, it was found that after being ingested by cancer cells, CAFs-derived exosomes could inhibit mitochondrial OXPHOS, thereby increasing glycolysis and glutamine-dependent reductive carboxylation in prostate cancer [174]. Importantly, a study based on proteomic profiling and functional dissection revealed that exosomes derived from CRC cells at different stages exhibited different roles in generating phenotypically and functionally distinct subsets of CAFs by reprogramming their proteome [175].

Cancer cachexia is a devastating, multifactorial and often irreversible syndrome that affects around 50–80% of cancer patients and accounts for up to 20% of cancer deaths [176]. It is characterized as substantial weight loss, primarily from loss of body fat and skeletal muscle, which are subjected to regulation in large part by exosomes-mediated signaling [177, 178]. It was revealed by Sagar et al. that exosomes derived from PCs could promote lipolysis in adipocytes via enhanced phosphorylation of hormone-sensitive lipase (HSL) by adrenomedullin (AM)/AM receptor (ADMR)-activated ERK1/2 and p38 MAPK pathways [179]. Another study indicated that breast cancer cells-secreted exosomal miR-204-5p induced leptin signaling pathway in white adipose tissue (WAT) by targeting VHL, which resulted in fat loss and cancer-associated cachexia [180]. Skeletal muscle loss, being one of the most obvious and main symptoms of cachexia, is mainly caused by enhanced proteolysis through two most important cellular degradation systems, the ubiquitin proteasome and autophagy lysosome [181, 182]. Furthermore, two miRNAs enriched in exosomes of CRC cells, miR-195a-5p and miR-125b-1-3p, were found to induce skeletal muscle wasting by targeting Bcl-2-mediated apoptosis [183]. Study from the same research group further demonstrated that growth differentiation factor 15 (GDF-15) presented in CRC cells-derived exosomes could also directly induce apoptosis of myocytes via regulating Bcl-2/caspase-3 pathways [184]. Collectively, these studies suggested that cargoes in cancer cells-derived exosomes play important roles in development of cachexia and may act as valuable therapeutic targets in exploring novel treatment in the future [185, 186].

#### Exosomes-Mediated Regulation of Tumor Immunology

Although long-realized, tumor immunology can actually be regarded as an emerging field [187]. During the past two decades, immune system has been increasingly recognized as an important player in control or contribute to development of cancer [188, 189]. Initially hypothesized by Schreiber et al., it has now been widely accepted that cancer immunoediting, a process consisting of three sequential phases termed elimination, equilibrium, and escape, involves in cancer evolution [190]. In the phase of elimination, transformed cells can be directly destroyed by the intact immune system. Few cancer cells with the capability of surviving elimination can progress into the equilibrium phase, in which the adaptive immune system prevents outgrowth of cancer and also reshapes the immunogenicity of the cancer cells. The escape phase is described as progressively growing of cancer after successfully acquiring the ability to circumvent immune recognition and/or destruction [191]. In general, cancer cells enabled with characteristics such as genomic and epigenetic instability can escape the immune system through many different mechanisms involving loss of antigens, reduced antigen presentation, as well as establishment of an immunosuppressive state within the tumor microenvironment [192, 193]. It is believed that exosomes play a critical role in mediating immunosurveillance and cancer immunoediting [194]. Since the precise roles and underlying mechanisms of exosomes in regulating tumor immunology have been well reviewed elsewhere [195–198], we chose not to discuss this field in more detail here, but rather to summarize some recent advances.

Natural killer (NK) cells are innate lymphoid cells involved in tumor surveillance. While the biological behavior of NK cells can be affected by exosomes derived from cancer cells, NK cell exosomes can also modulate the immune system or elicit anti-tumor effects against certain cancer [199, 200]. CD8^+^ T cells are known to be the end effectors of cancer immunity and most forms of effective cancer immunotherapy involve CD8^+^ T cell effector function [201]. T cells infiltrated into cancer tissue receive many extrinsic signals from the local microenvironment, and these signals shape T cell differentiation, fate and function [202]. Recently, while it was demonstrated by in situ tumour arrays that T cell infiltration is dynamically controlled in time and space by the tumour microenvironment [203], distinct spatiotemporal dynamics of CD8^+^ T cell-derived cytokines were evidenced to act as local or global modifier of TME or tumor tissue, respectively [204]. It was reported by Yang et al. that bladder cancer cell-derived exosomal circTRPS1 could modulate the intracellular ROS balance and CD8^+^ T cell exhaustion via the circTRPS1/ miR141-3p/GLS1 axis [205]. In HCC, the increased circCCAR1 levels were showed both in HCC cell lines and exosomes in the plasma of patients and evidenced to correlate with poor prognosis [206]. Exosomal circCCAR1 secreted by HCC cells could be taken in by CD8^+^ T cells and caused its dysfunction by stabilizing the PD-1 protein [206]. Moreover, Xu et al. showed that prostate cancer cell-derived exosomes could foster immune evasion by impeding the function of CD8^+^ T cells [207]. Mechanistically, tumor exosomal IL-8 was demonstrated to induce reprogramming of energy metabolism in CD8 + T cells through overactivating PPARα and uncoupling protein 1 (UCP1), thereby resulting in exhaustion of CD8^+^ T cells though enhanced starvation [207]. As sentinels of the immune system, dendritic cells (DCs) have been of great interest to immunologist owing to their capacity to process and present antigen. Our updated knowledge of role of DCs in cancer immunology indicates that the interactions between DCs and T cell do not only govern T cell priming in the lymph nodes (LNs), but also are critical throughout the cancer immunity cycle including key reactions within the TME that promote antitumor effector responses [208, 209]. While the ability of DCs to present antigens as well as their maturation could be impaired by tumor-derived exosomes via diverse of mechanisms which has been reviewed elsewhere [195, 196], a recent study by Cocozza et al. aroused us to re-evaluate the respective proportions and functions of tumor-derived exosomes due to their heterogeneity [210]. Myeloid-derived suppressor cells (MDSCs) are major components of the immune suppressive TME whose differentiation has been demonstrated to be regulated by exosomal miRNAs derived from cancer cells [211–214]. MDSCs can be classified into two main populations: granulocytic MDSCs (G-MDSCs) and monocytic MDSCs (M-MDSCs) [215]. Although previous studies have revealed that in the tumor site, immune-suppressive macrophages were mainly differentiated from M-MDSCs [216, 217]; however, Wang et al. provided new evidence demonstrating that G-MDSCs could promote the differentiation of M-MDSCs into M2 macrophages through releasing exosomal miR-93-5p [215].

With the deep deciphering of the underlying mechanisms of tumor escape, numerous immunotherapies were developed by manipulating the immune system to reactivate the antitumor immune response and overcome the pathways leading to escape [218]. In addition to those well-established immune treatments including monoclonal antibodies, the successful introduction of immune checkpoint therapy (ICT) and chimeric antigen receptor-modified T (CAR-T) cell therapy into clinical practice has profoundly revolutionized the field of cancer immunotherapy [219–222]. ICT is designed to block inhibitory signals of T cell activation using antibodies against either cytotoxic T lymphocyte-associated protein 4 (CTLA-4) or programmed death-1 **(**PD-1)/ programmed death-ligand 1 **(**PD-L1) [223]. In 2010, ipilimumab, a monoclonal antibody (mAb) targeting CTLA-4, was shown remarkable clinical efficacy and received Food and Drug Administration (FDA) approval for the treatment of melanoma in 2011 [224]. Thereafter, mAbs blocking PD-1 or PD-L1 have received FDA approvals to treat a number of tumor types alone and in combination with other agents. Despite the unprecedented improvement in the durability of clinical responses with ICT, these responses are observed in only 20–30% of treated patients [220]. Low response rates to ICT can be attributed to a variety of mechanisms, among which the exosomes-remodeled TME is increasingly recognized as a pivotal player in determining the patient’s response upon treatment with ICT [225]. Successful presentation of tumor antigens by class I human leukocyte antigens (HLA-I/MHC-I) is an essential prerequisite for eliciting anti-tumour response [226]. Consequently, tumours have developed various means to limit HLA-I presentation of antigens via either genetic or epigenetic alterations, thus evading immune recognition. By using a long-read sequencer, a substantial frequency of mutations in *HLA-A*, *HLA-B*, and *HLA-C* were revealed in microsatellite instability-high (MSI-H) colorectal cancers [227]. The accumulation of mutations was shown to account for the reduced expression of HLA-ABC genes and contribute to the immune evasion [227]. In addition to genetic and epigenetic changes, the recent discovery of epitranscriptomics has added another layer of dynamic gene regulation [228]. *N*^6^-methyladenosine (m6A) is the most common and abundant endogenous modification in eukaryotic RNAs whose biological function in cancer development has attracted much attention currently [229]. While tumor-intrinsic YTHDF1, a versatile and powerful m6A reader, was demonstrated to drive immune evasion and resistance to immune checkpoint inhibitors via promoting MHC-I degradation, an exosome-mediated CRISPR/Cas9 system was shown to introduce YTHDF1 deficiency and restore expression of MHC-I and tumor immune surveillance [230]. Additionally, M2 macrophage-derived exosome was revealed to confer cancer cell resistance to ICT via apolipoprotein E (ApoE)-mediated downregulation of MHC-I [231]. Upregulated expression of PD-L1 on surface of tumor cells is a common strategy hijacked by various type of cancer to evade immune surveillance [232]. Moreover, elevated expression of PD-L1 and/or altered expression of PD-L1-targeted miRNAs in exosomes derived from tumor cells were also observed in several studies [233–235], suggesting that they may act as either potential therapeutic targets or biomarkers in ICTs [236, 237]. The DNA methylation and histone modification, two main epigenetic alterations, contribute to the acquisition of hallmark tumor capabilities by regulating gene expression programs that promote tumorigenesis [238, 239]. Accumulating evidence has shown that altered DNA methylation plays a crucial role in remodeling tumour immune microenvironment (TIME) and is associated with the response to ICIs [240, 241]. Since a global methylation loss has been demonstrated to correlate with tumor immune evasion signatures independently of mutation burden and aneuploidy [242], and the CD8^+^ T cell responsiveness to anti-PD-1 in melanomas was also demonstrated to be epigenetically regulated by histone methyl transferase Suv39h1 [243]. Thus, whether exosomes may participate in these processes is of particular interest and further investigation is in urgent need.

The adoptive transfer of CAR-T cells against CD19 was effective in treating relapsed and refractory acute lymphoblastic leukemia (ALL) with complete remission rates of up to 90% [244]. However, only limited efficacy of CAR-T therapy against other cancer types, especially solid tumours, was achieved [245]. When applied in the treatment of patient with solid tumours, CAR-T therapy is now facing a unique set of challenges including a lack of robustly expressed, tumour exclusive antigen targets as well as highly immunosuppressive TME [246]. A recent report by Zhong et al. indicated that solid tumors could release exosomes carrying both targeted tumor antigens and PD-L1, which acting as cell-free functional units to preferentially interact with cognate CAR-T cells and efficiently inhibited their proliferation, migration, and function [247]. Thus, this study provided a molecular explanation for CAR-T therapy resistance and suggested that strategies targeting exosome secretion may enhance CAR-T cells efficacy. On the contrary to the role of metabolic reprogramming in fostering the growth of cancer cells, metabolic deregulation and imbalance in immune cells within the TME have been reported to drive immune evasion and to limit therapeutic outcomes [248]. Given the manifold roles of exosomal cargoes in mediating metabolic reprogramming in TIME [171], it is worth noting that simultaneous targeting of dysregulated metabolic reprogramming may help to improve the response to cancer immunotherapy and deserves further exploration. Actually, a recent study by Hernández-López et al. has already demonstrated that dual targeting of cancer metabolome and stress antigens (such as NKG2D ligands or CD277) could affect transcriptomic heterogeneity of engineered T cells and improve their efficacy [249].

#### Exosomes-Mediated Regulation of Cancer Metastasis

Metastasis remains the leading cause of cancer-related deaths and is responsible for as much as 90% of cancer-associated mortality [250]. The development of metastasis involves in a series of biological events collectively termed the metastatic cascade, which can be categorized into three phases including dissemination, dormancy, and colonization, with each phase being tightly regulated by genetic as well as epigenetic changes of tumor cells themselves and crosstalk between tumor cells and TME [251–253].

A compelling body of evidence has indicated that exosomes participate in regulation of metastatic cascade of cancer through inducing phenotypic plasticity of cancer cells, reshaping TME and distant pre-metastatic niches (PMNs) [254, 255]. In primary tumor, some tumor cells undergo epithelial-mesenchymal transition (EMT) to acquire increased ability of migration and invasiveness, which marking the first step of metastasis [256]. Multiple aberrant signaling pathways, such as transforming growth factor β (TGF-β) and RTK, govern EMT through regulating a core set of EMT-activating transcription factors (EMT-TFs), which include SNAI1, Slug/SNAI2), Twist-related protein 1 (TWIST1), ZEB1/2 [257, 258]. It has been well documented that the EMT can also be driven by the coordinated and dynamically regulated functions of signaling molecules, miRNAs, and EMT-TFs carried in exosomes [254, 259–261]. While M2 macrophage infiltration was revealed to correlate with metastasis and a poor prognosis in patients with HCC by Lu et al., their mechanistical study further suggested that M2 macrophage-derived exosomal miR-23a-3p plays a central role in enhancing HCC metastasis by promoting EMT and angiogenesis, as well as increasing vascular permeability [262]. In contrast, as revealed by Luo et al., circPOKE, a circRNA downregulated in primary and metastatic breast cancer tissues, could suppress EMT of cancer cells via inhibiting the ubiquitin-specific peptidase 10 (USP10)-Snail axis [263]. Upon phenotypic change via EMT, tumour cells can subsequently intravasate into the blood or lymphatic system. After entering the circulation, circulating tumour cells (CTCs) manage to survive and travel to target sites, where they can extravasate, proliferate and establish metastatic lesions [264]. The comprehensive roles and mechanisms of exosomes on CTCs during tumor metastasis, including the ability of exosomes to enhance the shedding of CTCs, protect CTCs in circulation and determine the direction of CTC metastasis, can be referred to a recent review [265].

It has been well established that organs of future metastasis are not passive receivers of CTCs, but are instead selectively and actively reshaped by the primary tumour before metastatic spread has even occurred [266]. The formation of these predetermined microenvironments termed PMNs is associated with local changes including remodeling stroma and ECM, increasing vascular permeability, shaping of the local inflammatory and immunosuppressive microenvironment, and inducing metabolic reprogramming, all of which have been demonstrated to be regulated in part by exosomes [255, 267, 268]. While early dissemination of HER2-positive breast cancer cells to lung was mainly driven by the intrinsic characteristics of primary lesions [269, 270], the formation of PMNs is prerequisite for successful seeding of disseminated cancer cells (DCCs) and subsequent development of metastases [271]. It was recently revealed that breast cancer cells-derived exosomal caveolin-1 (Cav-1) could promote ECM deposition in lung fibroblasts as well as inhibit the PTEN/CCL2/VEGF-A signaling pathway in lung macrophages, thus contributing to the PMN formation [272]. The proceeding from ECM remodeling to immunosuppression represents a key and relatively complicate step in PMN formation [273]. It was reported that tumor‑derived exosomes were able to promote the secretion of CCL1 by lung fibroblasts, which in turn resulted in differentiation of regulatory T cells (Tregs) via activating its specific receptor CCR8 and establishment of an immunologically tolerant PMN [274]. Moreover, two independent studies have uncovered that tumor-derived exosomes can induce upregulation of PD-L1 through different mechanisms to drive polarization of lung immunosuppressive macrophages, thereby promoting the formation of immunosuppressive PMN [275, 276]. In addition to their effects on cellular components of immune microenvironment, tumor-derived exosomes were also demonstrated to activate alveolar epithelial Toll-like receptor 3 (TLR3) to recruit neutrophils, which led to formation of lung PMN [277]. Research into the immunomodulatory roles of lung epithelial cells by the same group further revealed that the expression of glutathione peroxidase 3 (GPX3) was significantly increased in tumor exosomes-educated alveolar type 2 (AT2) epithelial cells. This subpopulation of GPX3^+^ AT2 cells was found to promote PMN formation through secreting interleukin (IL)-10 to suppress T cell function [278].

Liver is another tropic organ of metastasis of many types of cancer. Several seminal studies have shown that primary tumor-derived EVs/exosomes travel to the liver and facilitate PMN formation and pro-metastatic inflammatory responses [279–283]. As demonstrated by Wang et al. that fatty acid cargo of tumour extracellular vesicles and particles (EVPs)-particularly palmitic acid-could induce secretion of tumour necrosis factor (TNF) by Kupffer cells, generating a proinflammatory microenvironment, suppressing fatty acid metabolism and oxidative phosphorylation, and promoting fatty liver formation [284]. Furthermore, hepatocyte-derived EVs in fatty liver was showed to enhance the progression of CRC liver metastasis by promoting oncogenic YAP signaling and an immunosuppressive microenvironment [285]. Immunosuppression is a hallmark of PDAC which contributes to early metastasis and poor patient survival. Substantial investigations have indicated that PDAC-secreted exosomes could induce the formation of PMNs in the liver, the most common site for PDAC metastasis, through molecular and metabolic regulation of immunosuppression [286, 287].

The organ colonization of disseminated tumor cells (DTCs) is an inefficient and rate-limiting step of metastasis. Only a few DTCs evading immune surveillance and surviving in PMN ultimately colonize and give rise to metastases in the distant organs. To date, significant advances in metastasis research have revealed that metabolic reprogramming in PMN is essential for the development of metastatic lesions [266]. In breast cancer lung metastasis models, a higher pyruvate carboxylase (PC)-dependent anaplerosis was initially discovered in lung metastases as compared to primary breast cancers [288]. Pyruvate enriched in the lung interstitial fluid was then evidenced to be taken up by DTCs initiating a metabolic cascade to remodel the ECM that supports formation of a permissive lung metastatic niche [289]. As has been addressed above, exosomes-mediated metabolic reprogramming plays an equally important role in fueling the growth of both primary and metastatic tumors. However, it is increasingly clear that the scenario in terms of metabolic reprogramming in PMN is different from that in primary tumor in many facets. While overexpression of miR-122 was showed to reduce the growth of primary breast cancer by restricting glucose uptake, exosomal miR-122 derived from breast cancer cells could increase nutrient availability of tumor cells in the distant PMN through suppressing glucose uptake by non-tumor niche cells such as lung fibroblasts, brain astrocytes and neurons, thus facilitating metastasis [290]. Given the findings that neutral lipid accumulation in infiltrated innate immune cells within the lung microenvironment endows them with metastasis-promoting capacities contributing to formation of the pre-metastatic and metastatic niches [291, 292], Gong et al. reported that lung-resident mesenchymal cells (MCs) can also accumulate neutral lipids at the pre-metastatic stage of breast cancer induced by interleukin-1β (IL-1β) [293]. Importantly, lipid-laden lung MCs were demonstrated to foster breast cancer metastasis through metabolic reprogramming of tumor cells and NK cells mediated by exosome-like vesicles, which shed new light on the metabolic role of the distant organ environment in supporting metastasis of primary tumors [293].

Despite all of aforementioned essential roles of exosomes as communicator extraordinaire in mediating cancer metastasis [294], how dysregulated biogenesis of exosomes contribute to metastasis remains largely unknown. PTEN as a well-known tumor suppressor governing a variety of biological processes, its functions in cancer metastasis are also deeply deciphered [295, 296]. It has been demonstrated that PTEN could suppress proteasome activity through FOXO1-mediated transcriptional repression of proteasome subunits in both cholangiocarcinoma and gallbladder cancer [297, 298]. Interestingly, Jiang et al. unraveled a novel function of PTEN in mediating lysosome biogenesis and acidification, whereby PTEN deficiency promoted exosome release in a transcription factor EB (TFEB)-dependent manner and resulted in increased tumor metastasis in cholangiocarcinoma [299, 300]. GPR143, the protein product of the ocular albinism type 1 (*OA1*) gene, is an atypical G-protein coupled receptor (GPCR) mainly localized in intracellular organelles, such as late endosomes and melanosomes [301]. In a study by Lee et al., an unknown function of GPR143 in exosome biogenesis and the mechanisms for the regulation of ESCRT-dependent MVB formation was identified [302]. GPR143 was found to mediate the recruitment of ESCRT-0 proteins to endosomes and modulates interaction with endosomal protein cargo, which regulating protein sorting in ILVs and exosome formation containing distinct cargo. Consequentially, GPR143-ESCRT-dependent control of exosome biogenesis was demonstrated to promote cell motility and cancer metastasis via governing the exosomal proteome composition [302].

With the continued efforts made on dissecting the metastatic process, our understanding of the complexity of metastasis has been extensively expanded [303]. In 2013, a pioneer study published in *Science* demonstrated for the first time that nerve fibres could sprout into tumour tissues through the process of axonogenesis in mouse models of prostate cancer, where they could contribute to cancer growth and dissemination [304]. Thereafter, the discovery that tumour tissues are infiltrated by autonomic or sensory nerve fibres was confirmed in a wide range of malignancies as has been well reviewed [305–308], which marking the emerging of a novel field of cancer neuroscience [309]. Nerves can not only serve as a route of cancer spread [310], but also remodel lymph vasculature to promote tumour cell dissemination [311]. Moreover, neurons and glial cells communicate directly with malignant cells or other cellular components in the TME through paracrine factors and, in some cases, through neuron-to-cancer cell synapses, to promote metastasis [312, 313]. To delineate the mechanisms of cancer dissemination along nerves, it has been proposed that the nerve-cancer cell crosstalk in perineural niche should be considered part of the TME while exploring [314, 315]. Actually, a series of studies on breast cancer have offered an exemplification in supporting of this notion. Among all breast cancer subtypes, triple-negative breast cancer (TNBC) has the highest incidence of central nervous system (CNS) metastasis, affecting nearly 30% of patients [316]. TNBC tumors are innervated and often express β2-adrenoceptor (β2AR) [317, 318]. While preclinical studies have shown that activation of β2AR signaling in TNBC cells can remodel the actin cytoskeleton and stimulate invadopodia formation to promote tumor cell invasion and metastasis [319, 320], genetic silencing of β2AR in TNBC cells was demonstrated to reduce metastasis [321]. In xenograft mouse models of TNBC, treatment with anthracycline was evidenced to increase sympathetic nerve fiber activity and norepinephrine concentration in mammary tumors through the induction of nerve growth factor (NGF) by tumor cells, thus driving metastasis [322]. Cancer exosomes have been demonstrated to induce tumor innervation [323], and a reciprocal feedback between colon cancer cells and schwann cells mediated by exosomes were also reported to promote the proliferation and metastasis of colon cancer [324]. More recently, neurotransmitter gamma-amino butyric acid (GABA) induced by sleep deprivation was shown to promote the metastasis of colon cancer through miR‑223‑3p endogenous pathway and exosome pathway [325]. Thus, it is anticipated that elucidating the precise exosomes-mediated mechanisms underlying nerve-driven cancer metastasis may advance effective therapies for patients with neurotropic cancers [326].

#### Exosomes-Mediated Resistance to Cancer Therapy

In the past decades, with the rapid progress of multi-omics analysis as well as development and use of elegant preclinical models, our understanding of cancer has profoundly advanced [303, 327]. In parallel with these advances, novel mechanism-based therapies such as targeted therapy and immunotherapy have been substantially developed and come into clinical practice [328, 329]. Although patients with cancer benefit a lot from currently available treatment, the primary (intrinsic) or secondary (acquired) resistance to therapy remains an obstacle to be overcome when pursuing better treatment outcome [330].

Till now, chemotherapy is still the most common and widely used anti-cancer therapy. However, cancer cells develop versatile of mechanisms, both genetic and non-genetic, to gain resistance to chemotherapeutics, which resulting in limited clinical efficacy [331, 332]. In addition to altered drug activity, decreased uptake and/or increased efflux of drugs, it has been increasingly recognized that evasion of drug-induced RCD including apoptosis and ferroptosis plays vital role in determining chemo-resistance in the clinical and experimental setting [333, 334]. In fact, a mounting body of evidence suggests that exosomes involve in almost all of previously recognized mechanisms of chemo-resistance [335]. In epithelial ovarian cancer (EOC), exosomal miR-6836 derived from cisplatin-resistant EOC cells was demonstrated to increase stemness and suppress apoptosis in recipient cell by targeting DLG2-YAP1 signaling pathway, thus transferring chemoresistance phenotype [336]. Emerging studies have indicated that EVs-mediated ferroptosis can regulate the therapeutic responses of tumours [337–339]. As has been mentioned above, the Exo-ACADM can promote the gemcitabine-resistance in PCs through not only metabolic reprogramming, but also affecting ferroptosis via GPX4 and mevalonate pathways [166]. The similar ferroptosis-related mechanism of therapeutic resistance was further confirmed by Qi et al. whose study demonstrating that CAFs could suppress ferroptosis and induce gemcitabine-resistance in PCs by secreting exosome-derived ACSL4-targeting miRNA [340]. Interestingly, however, a CAFs-derived exosomal lncRNA DACT3-AS1 was showed to confer sensitivity of GC cells to oxaliplatin through SIRT1-mediated ferroptosis, suggesting that exosome-mediated induction of ferroptosis might be a promising strategy to reverse drug resistance [341, 342]. Autophagy is an important cellular degradation pathway that can be triggered by chemotherapeutics and provide the essential nutrients to rapidly dividing cancer cells. Studies have uncovered that there exists an extremely complex crosstalk between autophagy and exosome. While exosomes secreted by cancer cells can modulate autophagy in recipient cells, autophagy can influence the biogenesis of exosomes [343]. Given the function of autophagy as either a pro-survival or a pro-death phenomenon, its role in determining drug sensitivity is dual [344]. It was revealed that a novel protein encoded by exosomal circATG4B, circATG4B-222aa, could induces oxaliplatin resistance in CRC by promoting autophagy [345]. In a report from Zhang et al., exosome-mediated transfer of lncRNA SNHG7 was found to enhance docetaxel resistance in LAUD through promoting autophagy via recruiting human antigen R (HuR) to stabilize autophagy-related genes autophagy related 5 (ATG5) and ATG12 [346]. However, also in CRC, CAFs-derived exosomal lncRNA FAL1 was evidenced to act as a scaffold for the interaction between Beclin1 and TRIM3 to promote TRIM3-dependent Beclin1 polyubiquitination and degradation, thereby suppressing oxaliplatin-induced autophagic cell death [347]. Furthermore, in response to epirubicin (EPI), the ROS was increased in CAFs and triggered autophagy, while transcription factor 12 (TCF12) could inhibit autophagy flux and further promote exosome secretion as has been demonstrated by Qiu et al. [348]. Mechanistically, exosome secreted from EPI-treated CAFs was showed to not only prevent ROS accumulation in CAFs but also upregulate the CXCR4 and c-Myc protein levels in recipient cells, thus promoting EPI resistance of ER + breast cancer [348].

Cumulative evidence has indicated that induced EMT not only endows cancer cells with increased ability of migration and invasiveness, but also confers to acquired chemoresistance in various cancer types [349, 350]. It has been previously unveiled that CAFs-derived exosomal miR-92a-3p could activate Wnt/β-catenin pathway and inhibit mitochondrial apoptosis by directly inhibiting FBXW7 and MOAP1, which in turn contributed to cell stemness, EMT, metastasis and 5-FU/L-OHP resistance in CRC [351]. Recently, it was demonstrated that CAFs-derived exosomal LINC00355 could also promote EMT and chemoresistance in CRC through the miR-34b-5p/CRKL axis [352]. Conversely, Xu et al. confirmed that exosomal miR-451a derived from human umbilical cord MSCs (hUCMSCs) could inhibit the EMT of HCC cells via targeting ADAM10, thus overcoming the paclitaxel (PTX)-resistance in HCC [353]. CSCs are a special subset of cancerous cells contributing to tumor initiation, metastasis, relapse and chemoresistance [354]. There emerges an opinion that CSCs can originate from dedifferentiation of cancer cells driven by EMT [355]. This notion was supported by Hu et al. whose study confirming that CAFs-derived exosomal Wnt could induce dedifferentiation of CRC cells to phenotypic and functional CSCs, which conferring CRC chemoresistance [356]. Other exosomal cargos including lncRNA H19 [357] and circ_0001610 [358] were also reported to promote stemness and chemoresistance of CRC through different mechanisms. Moreover, in bladder cancer, CAFs-derived miR-146a-5p was demonstrated to foster CSC niche formation and cancer stemness to drive chemoresistance via co-targeting ARID1A and PRKAA2 (also known as AMPKα2) [89]. Given these findings, the therapeutic strategy to overcome drug resistance through targeting exosomes-induced EMT and CSCs is attracting much interest [47, 359].

Radiotherapy (or radiation therapy, RT) is one of the three traditional components of cancer treatment. It is estimated that approximately half of patients will receive RT at some point after a diagnosis of cancer [360]. With the rapid improvements in diagnostic imaging, treatment planning, and treatment delivery, contemporary RT has gained more accurate and precise treatment of diseased tissue and avoidance of healthy tissues [361]. However, cancer patients often develop resistance to RT via complex mechanisms which have been reviewed elsewhere [362–364], thus resulting in RT failure. Emerging studies have demonstrated that RT can influence the biogenesis and contents of exosomes secreted by cancer cells or other cellular components in TME, and radiation-derived exosomes can confer radioresistance and facilitate radiation-induced bystander effects [365, 366]. It was illustrated that low-dose radiation induced secretion of exosomes with a high level of circ-METRN in glioblastoma cells, which resulting in radioresistance through miR-4709-3p/GRB14/PDGFRα pathway [367]. While irradiated cell-derived exosomes were showed to promote radioresistance via the MAPK/Erk pathway [368], low-dose aspirin was demonstrated to inhibit release of exosomes induced by RT in breast cancer and enhance the RT sensitivity [369]. In esophageal squamous cell carcinoma (ESCC), elevated expression of lncRNA-NORAD in radioresistant ESCC cells was found to confer RT resistance via EEPD1/ATR/Chk1 signalling and by inhibiting pri-miR-199a1 processing and the exosomal transfer of miR-199a-5p [370]. Meanwhile, hypoxic tumour cell-derived exosomal miR-340-5p and angiopoietin-like 4 (ANGPTL4) were evidenced to confer radioresistance in ESCC and NSCLC by targeting KLF10/UV radiation resistance-associated gene (UVRAG) and inhibiting ferroptosis, respectively [371, 372]. Moreover, RT resistance can also result from exosomes derived from other components in TME like MSCs, CAFs, and TAM [373–376].

Deregulated cancer-driven signalings have offered abundant druggable targets for cancer therapy. Since the first approval of therapeutic antibody Rituximab in 1997 [377], a surge in antibody- as well as small molecular inhibitor-based targeted therapy has improved outcomes in a wide range of cancers [378]. Even though these advances, a substantial number of patients develop resistance to targeted therapy and disease relapse [379]. The arise of resistance to targeted therapy can be different depending on the action of drug. Lung cancer is the most frequent cause of cancer-related deaths worldwide with NSCLC being the most prevalent subtype [380]. So far, targeted therapy for NSCLC has received extensive exploring and dozens of targeted drugs have been approved for the treatment of patients with NSCLC [381]. Osimertinib, a novel mutant-selective irreversible third-generation EGFR tyrosine kinase inhibitor (TKI), exhibits potent anticancer activity in EGFR-mutated (mutEGFR) NSCLC patients harboring the T790M gatekeeper mutation [382]. It was demonstrated that treatment with osimertinib could promote the formation and release of wild type EGFR (wtEGFR)-harbouring exosomes in wtEGFR-expressing NSCLC cells by upregulating a Rab GTPase (RAB17), and exosome-mediated intercellular transfer of wtEGFR further triggered osimertinib resistance in mutEGFR NSCLC through activating downstream PI3K/AKT and MAPK signaling pathways [383]. In addition, exosomes derived from M2 type TAMs were also showed to confer osimertinib resistance in NSCLC through MSTRG.292666.16‑​miR‑6836‑​5p‑MAPK8IP3 axis, suggesting that targeting TAMs may help to circumvent resistance of EGFR-TKIs [384, 385]. The monoclonal antibody trastuzumab is the first HER2-targeted drug approved for the treatment of patients with HER2-positive breast cancer [386]. Through binding to Hu antigen R (HUR), an exosomal lncRNA Linc00969 was evidenced to promote the mRNA stability and protein expression of HER2, thereby inducing trastuzumab resistance in HER2-positive breast cancer [387]. Sunitinib, a targeted therapeutic used in the treatment of renal cell carcinoma (RCC), is a TKI that inhibits the kinase activity of a number of RTKs including VEGFR [388]. It has been demonstrated that exosome-transmitted lncARSR could promote sunitinib resistance in RCC by acting as a competing endogenous RNA [389]. As the strategies as well as drugs of targeted therapy continue to emerge, the field of mechanisms underlying resistance to targeted therapy is expanding and deserved further investigation.

There is no doubt that the renaissance of tumor immunology has promoted the coming age of tumor immunotherapy and improved the outcome of patients with advanced cancer [219]. Just as almost all treatments of cancer except for surgery, the accompanying resistance to tumor immunotherapy is an inevitable obstacle to be overcome currently [390, 391]. As aforementioned above, many exosomes-based mechanisms underlying evasion of immune surveillance confer to resistance to immunotherapy including ICT [206, 230, 231], and CAR-T therapy in cancer as well [247]. In addition, reshaped TME resulted from conventional treatment such as radiotherapy, as well as ICT has also been increasingly recognized as key player in development of acquired resistance to cancer immunotherapy [392, 393], which adding new dimension to the field of even complicated exosome-mediated mechanisms [394].

**Fig. 1:**
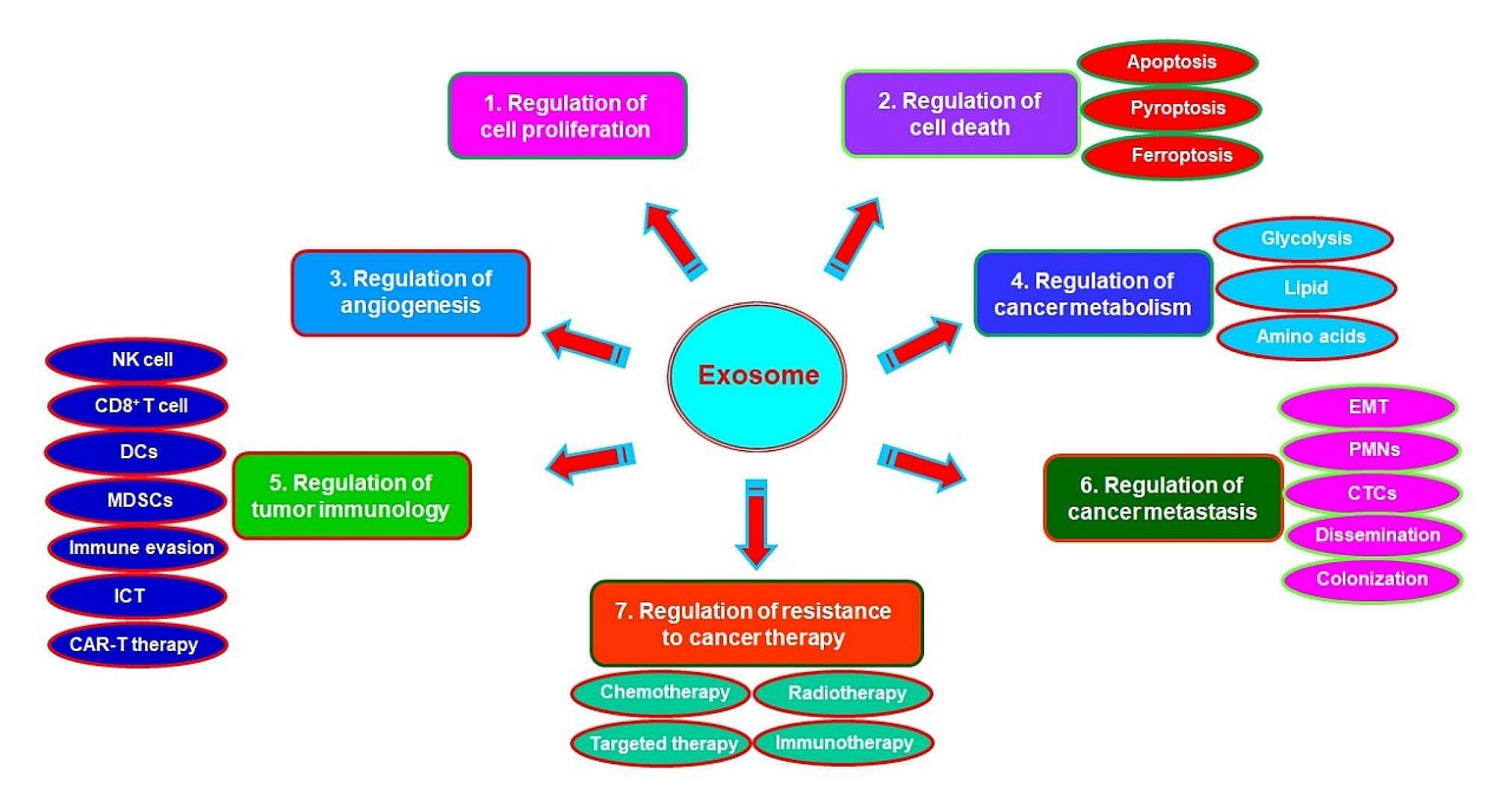
Role of exosomes in progression of cancer

Collectively, the roles of exosomes in progression of cancer are depicted in Fig. 1 and summarized in Table 3.

**Table 3:** Role of exosomes in progression of cancer

Exosomes source	Cargo	Key findings	Reference
**Regulation of cell proliferation**			
Glioma cell	EGFRvIII	Activate downstream AKT signaling in recipient cell	[99]
BC cell	EGFR, HER2	Activate MAPK signaling to promote monocyte survival in the inflammatory niche	[100]
GC cell	Undetermined	Promote the proliferation of GC cell by activating AKT and MAPK signalings	[101]
CAFs	miR-3656	Promote the development and progression of ESCC by activating AKT signaling	[102]
CAFs	miR-20a	Promote the progression of NSCLC through targeting PTEN	[105]
**Regulation of cell death**			
M2 TAMs	miR-21	Suppress apoptosis of GC cell via targeting PTEN	[110]
BC cell	tRF-16-K8J7K1B	Reduce drug-induced cell apoptosis by targeting TRAIL	[113]
Melanoma cell	miR-211-5p	Inhibit pyroptosis in recipient cell	[118]
CAFs	miR-522	Suppress ferroptosis in GC cell by targeting ALOX15	[121]
Adipocyte	MTTP	Suppress ferroptosis in CRC	[122]
Osteosarcoma cell	miR‑144‑3p	Promote ferroptosis in osteosarcoma by targeting ZEB1	[123]
**Regulation of angiogenesis**			
GC cell	miR-301a-3p	Inhibit HIF-1α degradation through targeting PHD3	[129]
Endothelial cell	VEGFA	Promote angiogenesis of HCC	[133]
CAFs	VEGFA	Promote angiogenesis of CRC	[134]
GC cell	circSHKBP1	Promote angiogenesis of GC by targeted regulating the miR-582-3p/HUR/VEGF axis	[135]
PC cell	miR-30b-5p	Promote angiogenesis of PC by inhibiting GJA1	[136]
OC cell	miR-205	Promote angiogenesis of OC	[137]
M2 TAMs	miR-155-5p, miR-221-5p	Promote angiogenesis of PC by targeting E2F2	[138]
MSCs	miR-100	Suppress in vitro angiogenesis of BC through modulating the mTOR/HIF-1α/VEGF signaling axis Regulation of cancer metabolism	[139]
**Regulation of cancer metabolism**			
MSCs	si-PYCR1	Inhibit aerobic glycolysis of BlC cell	[147]
GC cell	lncRNA CCAT1	Augment glycolysis in GC cell	[150]
BC cell	miR-105	Enhance glycolysis in CAFs to fuel adjacent cancer cell	[158]
BC cell	ITGB4	Induce mitophagy and glycolysis in CAFs	[159]
CRC cell	HSPC111	Promote CRC liver metastasis by reprogramming lipid metabolism	[163]
GC cell	lncAKR1C2	Promotes GC lymph node metastasis by regulating fatty acid metabolis	[165]
PC cell	ACADM	Promote the gemcitabine-resistance in PCs via increasing hydrolysis of medium-and long-chain fatty acids	[166]
M2 TAMs	miR-193b-3p	Promote glutamine uptake of PCs by targeting TRIM62	[172]
CAFs	LINC01614	Enhance the glutamine uptake in LUAD cell	[173]
CAFs	Metabolites	Provide metabolites to fuel the growth of PrC	[174]
PC cell	AM	Promote lipolysis in adipocytes	[179]
BC cell	miR-204-5p	Induce leptin signaling pathway in WAT	[180]
CRC cell	miR-195a-5p, miR-125b-1-3p	Induce skeletal muscle wasting by targeting Bcl-2-mediated apoptosis	[183]
CRC cell	GDF-15	Induce apoptosis of myocytes via regulating Bcl-2/caspase-3 pathways Regulation of tumor immunology	[184]
**Regulation of tumor immunology**			
NK cell	let-7b-5p	Inhibit cell proliferation of PCs by targeting CDK6	[200]
BlC cell	circTRPS1	Induce CD8+ T cell exhaustion	[205]
HCC cell	circCCAR1	Promote CD8+ T cell dysfunction	[206]
PrC cell	IL-8	Impede the function of CD8+ T cell	[207]
CCL cell	miR-155	Induce MDSCs	[213]
Glioma cell	miR-10a, miR-21	Induce MDSCs	[214]
G-MDSCs	miR-93-5p	Promote the differentiation of M2 TAMs	[215]
M2 TAMs	ApoE	Confer ICB resistance in GC	[231]
Melanoma cell	PD-L1	Contribute to immunosuppression	[233]
LAUD cell	miR-16-5p	Down-regulate PD-L1 expression	[234]
OC cell	miR-155-5p	Down-regulate PD-L1 expression in macrophages	[235]
PC cell	PD-L1	Inhibit the efficacy of CAR-T cell Regulation of cancer metastasis	[247]
**Regulation of cancer metastasis**			
BC cell	miR-200	Promote EMT of recipient cell	[260]
M2 TAMs	miR-23a-3p	Promote EMT and angiogenesis in HCC	[262]
BC cell	circPOKE	Inhibit EMT of BC cell	[263]
Cancer cell	Cargos	Regulate CTCs Reviewed in	[265]
BC cell	Cav-1	Promote formation of PMNs in lung	[272]
Lung carcinoma cell	Undetermined	Promote formation of immunologically tolerant PMNs in lung	[274]
NSCLC	Undetermined	Drive immunosuppressive macrophages in PMNs	[275]
GC cell	miR-92a‐3p	Facilitate lung PMNs formation	[276]
NSCLC	snRNAs	Promote lung PMNs formation	[277]
PC, GC, BC, CRC cell	MIF, Interigin, EGFR, miR-25-3p, miR‑519a‑3p	Promote liver PMNs formation	[279–283]
Hepatocyte	miR-25, 92, 103	Promote a metastatic tumor microenvironment	[285]
PC cell	miR-301a	Promote metastasis via M2 TAMs polarization	[287]
BC cell	miR-122	Promote metastasis via reprogramming glucose metabolism in PMNs	[290]
		metabolism in PMNs	
MCs	Lipids	Foster BC metastasis via metabolic reprogramming	[293]
GABA-induced CRC cell	miR‑223‑3p	Promote migration of CRC cell Regulation of resistance to cancer therapy	[325]
CRC cell			
**Regulation of resistance to cancer therapy**			
OC cell	miR-6836	Confer to cisplatin-resistance	[336]
CAFs	miR-3173–5p	Suppress ferroptosis and induce gemcitabine resistance in PC cells	[340]
CAFs	DACT3-AS1	Confer sensitivity of GC cells to oxaliplatin through SIRT1-induced ferroptosis	[341]
CRC cell	circATG4B	Induces oxaliplatin resistance in CRC by promoting autophagy	[345]
LAUD cell	SNHG7	Enhance docetaxel resistance in LAUD through promoting autophagy	[346]
CAFs	LINC00355	Promote EMT and chemoresistance in CRC	[352]
CAFs	LncRNA H19	Promote the stemness and chemoresistance of CRC	[357]
Glioblastoma	circ-METRN	Promote radioresistance in glioblastoma	[367]
ESCC cell	miR-340-5p	Promote radioresistance of ESCC	[371]
NSCLC	ANGPTL4	Promote radioresistance of lung cancer by inhibiting ferroptosis	[372]
CAFs	DNM3OS	Confer radioresistance of ESCC	[374]
M2 TAMs	circ_0001610	Reduce radiosensitivity in endometrial cancer	[375]
NSCLC cell	wtEGFR	Transfer targeted therapy resistance	[383]
M2 TAMs	ceRNA	Promote targeted therapy resistance in NSCLC	[384]
BC cell	Linc00969	Induce trastuzumab resistance in BC	[387]
RCC cell	LncARSR	Promote sunitinib resistance in RCC	[389]

EGFR: epidermal growth factor receptor; BC: breast cancer; HER2: human epidermal growth factor receptor-2; MAPK: mitogen-activated protein kinase; ESCC: esophageal squamous cell carcinoma; PTEN: phosphatase and tensin homolog deleted on chromosome 10; NSCLC: non-small cell lung cancer; TAMs: tumour-associated macrophages; GC: gastric cancer; tRFs: tRNA-derived fragments; TRAIL: tumor necrosis factor-related apoptosis-inducing ligand; ALOX15: arachidonate lipoxygenase 15; CRC: colorectal cancer; ZEB1: zinc-finger E-box-binding homeobox 1; HIF-1α: hypoxia-inducible factor 1α; PHD3: prolyl-hydroxylase 3; HCC: hepatocellular carcinoma; PC: pancreatic cancer; OC: ovarian cancer; MSCs: mesenchymal stem cells; PYCR1: pyrroline‑5‑carboxylate reductase 1; BlC: bladder cancer; ITGB4: integrin beta 4; ACADM: acyl-CoA dehydrogenase; TRIM62: tripartite motif (TRIM)-containing protein 62; LUAD: lung adenocarcinoma; PrC: prostate cancer; AM: adrenomedullin; WAT: white adipose tissue; GDF-15: growth differentiation factor 15; NK: natural killer; CDK6: cyclin dependent kinase 6; CLL: chronic lymphocytic leukemia; MDSCs: myeloid-derived suppressor cells; ApoE: apolipoprotein E; ICB: immune checkpoint blockade; EMT: epithelial-mesenchymal transition; CTCs: circulating tumor cells; Cav-1: caveolin-1; PMNs: pre-metastatic niches; snRNAs: small nuclear RNAs; MIF: migration inhibitory factor; MCs: mesenchymal cells; GABA: gamma-amino butyric acid; RCC: renal cell carcinoma

## Aptamer-Modified Exosomes in Cancer Targeted Therapy

Given the versatile roles of exosomes in initiation and malignant progression of cancer, exosomes themselves are actually emerging as candidate targets in cancer therapy [395]. Notably, owing to their biological properties such as excellent biocompatibility, ability to carry a wide range of bioactive cargos, and capability of being easily engineered or modified, exosomes as promising targeted drug delivery systems are of great interest to the scientific community and have been extensively explored in recent years [396–398]. The most common strategies to achieve targeted delivery of therapeutics by exosomes involve actively guiding the exosomes to target cells via functionalizing with antibodies or aptamers that can bind specifically to their corresponding antigens (targets) on recipient cells [399].

Aptamers, also known as chemical antibodies, are endowed with many superiorities over conventional antibodies in terms of low immunogenicity and cost, ease of synthesis, high binding specificity and affinity, as well as deeper tumor penetration [12]. Thus, aptamers as excellent targeting ligands for functionalizing exosomes have attracted extensive interest in cancer theranostics [12, 13, 400]. AS1411 is a well-known 26-base guanine-rich oligodeoxyribonucleotide aptamer that forms a G-quadruplex structure with many advantageous characteristics to bind specifically to nucleolin [401]. Nucleolin is a multifunctional protein localizing in the nucleolus, nucleoplasm, cytoplasm and cell membrane, which has been found to be overexpressed in a variety of cancers [402]. Thus, a large number of AS1411-based strategies have been developed in cancer targeted therapy so far [403]. Through conjugating with AS1411, the nucleolin-targeted exosome-mimetic extracellular nanovesicles (ENVs) loaded with chemotherapeutics paclitaxel were successfully developed by Wan et al. [404]. The paclitaxel-loaded AS1411-ENVs showed remarkable cancer treatment efficacy both in vitro and in vivo as evidenced [404]. AS1411-functionalized exosomes were also demonstrated by Hosseini et al. to achieve targeted delivery of doxorubicin (DOX) in fighting colorectal cancer [405]. TNBC is a more aggressive subtype of breast cancer lacking efficient targeted therapy currently. Based on the high expression of nucleolin in TNBC, an ingenious “triple-punch” strategy exosome-mimetic nanovesicles was successfully developed by Zhang et al. for TNBC targeted therapy guiding by aptamer AS1411 [406]. The AS1411-conjugated and DOX-loaded exosome-mimetic nanovesicles with overexpression of CD82 exhibited dual anti-cancer roles by inhibiting the metastasis and growth of TNBC. Recently, an AS1411-functionalized pH‑responsive biogenic titanium dioxide nanoparticles (TNP) was developed for targeted co-delivery of Forkhead box protein M1 (FOXM1) aptamer and DOX and enhanced therapeutic efficacy against breast cancer was demonstrated both in vitro and in vivo [407], suggesting that similar strategy may also be explored in developing aptamer-guided exosome delivery system. Moreover, it has been demonstrated that as compared with AS1411-modified lipid nanoparticles, the AS1411-modified exosomes exhibit better capability to escape from immune surveillance and longer circulation time, which making them an ideal in vivo targeted delivery system for cancer therapy [408].

In addition to chemotherapeutics, aptamer-functionalized exosomes have also been extensively exploited as targeted delivery system of nucleic acids including siRNA, miRNA, circRNA and mRNA for cancer therapy [409]. An EGFR-targeted aptamer CL4 was used in functionalizing exosomes to achieve targeted delivery of lncRNA DARS-AS1 siRNA to TNBC cells by Liu et al. [410]. Silencing DARS-AS1 was further demonstrated to attenuate DOX resistance in TNBC through suppressing the TGF-β/Smad3 signaling pathway-induced autophagy. Similarly, by using an EGFR RNA aptamer, exosomes preloaded with siRNA targeting Survivin, a negative regulator of apoptosis frequently overexpressed in NSCLC, were engineered in an attempt to achieve a specific gene knockdown effect in EGFR-positive NSCLC cells [411]. The siRNA delivery system was showed to suppress the growth of tumor cell as well as sensitize tumor cell to chemotherapy both in vitro and in vivo by downregulation of Survivin. SIRT6 is an important member of highly conserved family of NAD^+^-dependent histone deacetylase whose overexpression has been evidenced in many cancer types including prostate cancer [412]. As demonstrated, an aptamer-modified exosomes carrying SIRT6-targeted siRNA significantly inhibited the growth and metastasis of prostate cancer both in vitro and in vivo through silencing SIRT6 [413]. Previously, the therapeutic oligonucleotides are primarily loaded onto exosomes by encapsulation and surface modification; however, the loading efficiencies are relatively low [414]. Recently, an Exosomal Anchor DNA Aptamer (EAA) was obtained via SELEX against exosomes immobilized with CP05 peptides by Han et al. [415]. The EAA showed high binding affinity to different exosomes and enabled efficient loading of nucleic acid drugs on exosomes without complicated conjugation or modification, which providing a generalizable strategy for further developing exosomes-based delivery vehicles of nucleic acid drugs [415]. Interestingly, in addition to acting as guiding ligands, aptamers themselves have been explored as therapeutic acids which attracting much research interest [416]. Two aptamers targeting β-catenin and NF-κB were connected to construct artificial circular RNAs (acircRNAs) [417]. In vitro study indicated that after loading into exosomes via CD63-HuR fusion protein, the exosomes-delivered acircRNAs showed high potential to downregulate the expression of β-catenin and NF-κB in bladder cancer cells and inhibit their malignant phenotype consequently [417].

Owing to the many aforementioned fundamental roles of exosomes in tumor immunology, engineered exosomes have been substantially explored as next generation immunotherapeutics in these years [418]. Coxsackievirus B3 (CVB3) is a single-stranded RNA virus with broad anti-tumor activity [419]. Bahreyni et al. have previously reported that the miRNA-modified coxsackievirus B3 (miR-CVB3) could inhibit the growth of TNBC with improved safety profile in immunocompetent mice [420]. In a recent study from the same research group, infection with miR-CVB3 was demonstrated to reshape immune-related protein profiles in breast cancer [421]. Moreover, miR-CVB3 was evidenced to be present in exosome derived from infected cancer cell. Notably, after being engineered with the AS1411 aptamer and DOX, the so-called exosome, ExomiR-CVB3/DoxApt, exhibited enhanced antitumor cytotoxicity and bolstered immunostimulatory effects [421].

**Fig. 2:**
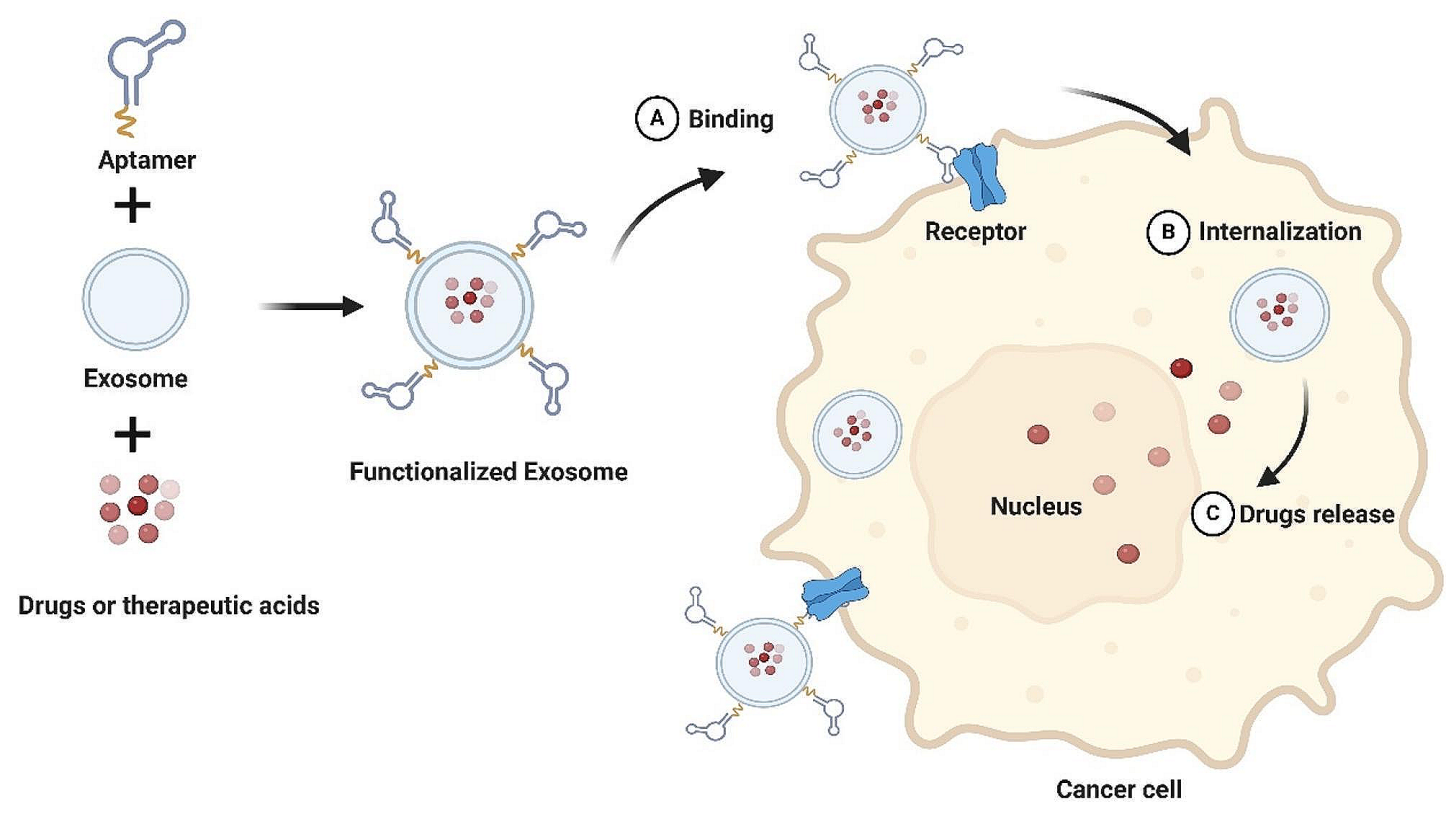
Diagram of aptamer-functionalized exosome in targeted therapy of cancer

To subvert the detrimental effect of CSCs on malignant progress and relapse of cancer, aptamers have been frequently applied in developing therapeutic strategies targeting CSCs [422]. Through conjugating with anti-CD20 aptamer, adriamycin-loaded exosomes were showed to hold a potential to target melanoma CSCs, which suppressing tumor growth both in vitro and in vivo [423]. Besides, aptamer-modified exosomes were also explored to targeted delivery of sonosensitizer indocyanine green, which further broadening their potential application in sonodynamic therapy against cancer [424]. Taken together, the aptamer-functionalized exosomes in cancer targeted therapy are concisely depicted in Fig. 2.

## Future Directions and Perspectives

Beyond question, elucidating the roles of exosomes in tumorigenesis and progression of cancer represents a rapid expanding field of cancer research currently. Although significant advances have been made in recent years, many critical unresolved issues need to be addressed. First of all, challenges in nomenclature, separation, and characterization of EVs including exosome remain hurdles in deepening the investigation from basic biology to clinical applications. Recently, the International Society for Extracellular Vesicles (ISEV) has updated its “Minimal Information for Studies of Extracellular Vesicles (MISEV2023)” to provide researchers with available approaches and their advantages and limitations for production, separation and characterization of EVs from multiple sources [425]. However, continued efforts are still needed to advance this field. Secondly, as the biogenesis of exosome is highly dynamic and the cargos vary in both type and concentration, fully unravelling the tumor biology of exosome is facing an insufficiency in tools and more exquisite in vivo models [426]. Thirdly, with the increasing recognition of link between the microbiota and cancer [427], it should be of great interest for research community to pay more attention to explore the role and precise underlying molecular mechanism of exosome in mediating the crosstalk between microbiota and cancer cell [428]. Finally, in terms of translating the aptamer- functionalized exosome into clinical practice, many challenges such as bioavailability, stability of exosomes and aptamers in vivo, as well as their potential side effects are facing now [429]. When applied in vivo, aptamers are susceptible to nuclease-mediated degradation that causes very short half-lives [430], thus strategies are in urgent need to improve their in vivo stability. Owing to few aptamer-based therapy has entered the clinical practice, the toxicological information regarding aptamers in humans is very limited currently. In addition, the source of exosome with safe should be taken into consideration [431]. In conclusion, even though our current understanding about the tumor biology of exosome is limited and the exploring the possible clinical application of aptamer-modified exosomes in cancer targeted therapy is preliminary, we still believe that further research into this field will not only broaden our comprehensive knowledge of tumor biology but also bring us novel exosome-based therapy against cancer in the near future.

## Data Availability

No datasets were generated or analysed during the current study.
